# *Schistosoma mansoni* rSm29 Antigen Induces a Regulatory Phenotype on Dendritic Cells and Lymphocytes From Patients With Cutaneous Leishmaniasis

**DOI:** 10.3389/fimmu.2018.03122

**Published:** 2019-01-09

**Authors:** Diego Mota Lopes, Sérgio Costa Oliveira, Brady Page, Lucas P. Carvalho, Edgar M. Carvalho, Luciana Santos Cardoso

**Affiliations:** ^1^Serviço de Imunologia, Hospital Universitário Professor Edgard Santos, Universidade Federal da Bahia, Salvador, Brazil; ^2^Instituto Nacional de Ciência e Tecnologia em Doenças Tropicais (DT/CNPq), Brasilia, Brazil; ^3^Departamento de Bioquímica e Imunologia, Instituto de ciências Biológicas, Universidade Federal de Minas Gerais, Belo Horizonte, Brazil; ^4^Massachusetts General Hospital, Boston, MA, United States; ^5^Instituto Gonçalo Moniz, Fundação Oswaldo Cruz, Salvador, Brazil; ^6^Departamento de Análises Clínicas e Toxicológicas, Faculdade de Farmácia, UFBA, Salvador, Brazil

**Keywords:** *Schistosoma mansoni*, cutaneous leishmaniasis, Sm29 antigen, dendritic cells, lymphocytes

## Abstract

The immune response induced by *Schistosma mansoni* antigens is able to prevent immune-mediated diseases. Conversely, the inflammatory response in cutaneous leishmaniasis (CL), although responsible for controlling the infection, is also associated with the pathogenesis of disease. The aim of this study was to evaluate the potential of the *S. mansoni* Sm29 antigen to change certain aspects of the profiles of monocyte derived dendritic cells (MoDCs) and lymphocytes from subjects with CL *in vitro*. Expression of surface molecules and intracellular cytokines in the MoDCs and lymphocytes as well as the proliferation of *Leishmania braziliensis* were evaluated by flow cytometry. Levels of cytokines were evaluated in culture supernatants by ELISA. It was observed that stimulation by rSm29 increased the frequency of expression of CD83, CD80, CD86, and IL-10R in MoDCs compared to non-stimulated cultures. Additionally rSm29 decreased the frequency CD4^+^ and CD8^+^ T cells expressing CD28 and increased the frequency of CD4^+^CD25^hi^ and CD4^+^CTLA-4^+^ T lymphocytes. Addition of rSm29 to cultures increased IL-10 levels and decreased levels of IL-12p40 and IFN-γ, while not altering TNF levels compared to non-stimulated cultures. This study showed that rSm29 induced a regulatory profile in MoDCs and lymphocytes and thereby regulated the exaggerated inflammation observed in CL. Considering that there are few therapeutic options for leishmaniasis, the use of rSm29 may be an alternative to current treatment and may be an important strategy to reduce the healing time of lesions in patients with CL.

## Introduction

Cutaneous leishmaniasis (CL) is an endemic disease in South and Central America, the Middle East, and Central Asia caused by a variety of *Leishmania* species. Considered a global public health problem, the disease has a high incidence with 1.3 million cases per year and ~200 million people living in high-risk areas (1). The various clinical manifestations of the disease are related to intrinsic factors of the parasite but also to factors related to the host immune response (2). The lesions are characterized by inflammation with a predominance of mononuclear cells and few or even no parasites present. The immune response is predominantly Th1-type, important for macrophage activation and parasite elimination. However, it is known that the Th1 immune response profile, if exaggerated, is the main cause of CL (3, 4). Thus, effector immunological mechanisms in CL lead to cellular and tissue destruction, culminating with the appearance of the characteristic ulcer that is the main clinical manifestation of CL. Therefore, it is important to have a balance between Th1 and regulatory T cells, since both of these mechanisms are important in maintaining the tissue integrity of the host against an exaggerated inflammatory response (5, 6).

Dendritic cells (DCs) play an important role in the interface between innate and adaptive immunity. They are recognized for their ability to sensitize naïve T lymphocytes and for contributing to the functional differentiation of regulatory T cells (7). Dendritic cells from the skin carry the parasite from the site of infection to the regional lymph nodes (8). During this migration, they differentiate into mature dendritic cells with potent antigen-presenting functions and the ability to activate T cells (9, 10). In this context, dendritic cells and lymphocytes play an important role in directing the immune response, mainly through the production of IFN-γ, TNF, and IL-12, which are important for macrophage activation and parasitic destruction (11, 12). However, other T cell subtypes have been described, such as regulatory T cells (Treg) and Th17 cells which also appear to play an important role in the susceptibility and resistance to CL (13–15).

Evidence has accumulated that chronic helminth infection, particularly by *Schistosoma* spp. or their products, is able to modulate the Th1-type inflammatory response involved in some immune-mediated diseases such as type I diabetes, rheumatoid arthritis, and HTLV-1 infection (16–19). A study conducted by Bafica et al. showed that the addition of *S. mansoni* rSm29 antigen in PBMC cultures of CL patients was able to decrease the production of TNF and IFN-γ and increase IL-10 production by these cells (20). Another study by Bafica et al. reported that monocytes from patients with CL stimulated by the rSm29 and soluble *Leishmania braziliensis* antigen (SLA) antigen had lower HLA-DR expression compared to SLA-stimulated cells only (21). Previous studies performed by our group showed that MoDCs from patients with CL when stimulated with rSm29 and SLA expressed more maturation (CD83) and costimulatory molecules (CD80 and CD86) in relation to non-stimulated cells or cells stimulated with SLA only (22). These unconventional activations of MoDCs have provided the rationale for the use of recombinant Sm29 antigen in *in vitro* studies with cells from patients with leishmaniasis infected by *L. brazileinsis* in an attempt to modulate the inflammatory response associated with pathogenesis.

## Materials and Methods

### Patient Selection

Included in this study were 12 patients with a diagnosis of CL, all of whom were residents of the endemic area of Corte de Pedra, Bahia, Brazil. The diagnosis of CL was made by a characteristic clinical picture of CL in addition to isolation of parasites from biopsy of the lesion or identification of amastigotes in histopathologic studies. All patients were evaluated prior to therapy. Three fecal samples from each individual were examined using the Hoffman sedimentation method to exclude helminth-infected individuals. To eliminate the effects of previous *S. mansoni* exposure in immunological assays, we also measured the levels of serum-specific IgE to *S. mansoni* soluble adult worm antigen (SWAP). All individuals evaluated were negative for helminth infections including *S. mansoni* infection or exposure to this parasite antigen. Among the individuals evaluated, 67% were male and 33% female, with a mean age of 33.5 ± 14.5 years. Of the 12 patients, 42% presented only one cutaneous lesion and 58% presented between two and four lesions.

### Ethics Statement

The present study was approved by the Ethics Committee of the University of the State of Bahia (License number 0603110287514) and informed written consent was obtained from all study participants. All participants were adults.

### Production of *S. mansoni* rSm29 Recombinant Antigen

The recombinant *Schistosoma mansoni* antigen used in this study, rSm29, was kindly provided by Dr. Sergio C. Oliveira of the Institute of Biological Sciences within the Department of Biochemistry and Immunology at the Minas Gerais Federal University. The gene encoding the rSm29 protein was cloned into a plasmid in competent *Escherichia coli* (23) strains and tested for the presence of lipopolysaccharide using a commercially available Chromogen Kit (LAL, CAMBREX). LPS levels were below the limit of detection (data not shown). Figure S1 shows the western blot analysis of purified recombinant Sm29.

### Preparation of Peripheral Blood Mononuclear Cells and Isolation of Monocytes and Lymphocytes

Peripheral blood mononuclear cells (PBMCs) of patients with CL were obtained using the Ficoll-Hypaque density gradient technique (GE Healthcare, Uppsala) and adjusted to a concentration of 1 × 10^7^ cells/mL in complete RPMI 1640 (100 μL/mL gentamicin, 2 mM L-glutamine, 30 mM HEPES) containing 10% inactivated fetal bovine serum (FBS) (Life Technologies GIBCO BRL, Gaithersburg, MD). Monocytes (CD14^+^) were isolated from PBMCs using magnetic beads (Monocyte Isolation Kit II, human, MACS, Miltenyi Biotec) by negative selection, using monoclonal antibodies (anti-CD3^+^, CD56^+^, CD16^+^, CD19^+^, and Glycophorin A^+^). Lymphocytes remaining after monocyte separation by magnetic beads were stored at −70°C in DMSO and 10% FBS for 7 days for further co-culturing with the MoDCs. The purity of monocyte and lymphocyte separation was determined by anti-CD14, CD11c, and CD3 monoclonal antibody labeling and evaluated by flow cytometry (Figure S2). Monocytes were CD14^+^ and CD3^−^ (Figure S2A). In addition, monocytes had low expression of CD11c (1.25%) (Figure S2A). To evaluate the purity of the lymphocytes, labeling with anti-CD3 and anti-CD14 antibodies was performed, where the selected population presented a mean frequency of 72% of CD3^+^CD14^−^ cells (Figure S2B). Monocytes were incubated with Fc blocking (FcR Blocking Human Reagent, Cat 120-001-571, MACS Miltenyi Biotec) prior to differentiation into dendritic cells.

### Differentiation of Monocyte-Derived Dendritic Cells and Stimulus With rSm29 or LPS

After separation, 2 × 10^6^ monocytes (CD14^+^) were incubated at 37°C in 5% CO_2_ for 7 days with 3 mL/well of complete RPMI containing 800 IU/mL IL-4 (R&D Systems) and 50 ng/mL GM-CSF (Peprotech) for differentiation into monocyte-derived dendritic cells (MoDCs). After 7 days of culture, the MoDCs were collected by washing the wells three times with saline and adjusted to the concentration of 3 × 10^5^ cells/mL for flow cytometry experiments. MoDCs were stimulated on the 6th day of monocyte differentiation with 10 μg/mL recombinant rSm29 or 100ng/mL *E. coli* O111:B4 (Sigma-Aldrich) LPS for 24 h at 37°C in 5% CO_2_.

### Culture of Leishmaniasis and Marking With CFSE

The strain of *L. braziliensis* (MHOM/BR/2007/LTCP11245) was obtained from lesion aspirates of a patient with CL and cultured initially in NNN biphasic medium (Neal, Novy, Nicolle from SIGMA-Aldrich) and later in Schneider media supplemented with penicillin, streptomycin, and fetal bovine serum. Metacyclic promastigotes were obtained from the culture in the stationary phase (~5–7 days) and were used to infect the MoDCs. Prior to infection, promastigotes were incubated with carboxyfluorescein diacetate succinimidyl ester (CFSE; Invitrogen) for further analysis of proliferation by flow cytometry (Figure S2C) as described by Kamau et al. (24).

### Infection of MoDCs With Promastigotes of *L. braziliensis* and Co-culture With Autologous Lymphocytes

After 24 h of stimulation by rSm29 or LPS antigens, 3 × 10^5^ MoDCs were cultured with 15 × 10^5^ *Leishmania* parasites for a ratio of 5 parasites to 1 MoDC. Cultures of MoDCs with the parasites were incubated for 2 h at 37°C in 5% CO_2_ and then washed twice with complete RPMI 1640 (100 μL/mL gentamicin, 2 mM L-glutamine, 30 mM HEPES) containing 10% inactivated Fetal Bovine Serum (Life technologies GIBCO BRL, Gaithersburg, MD) to block infection and remove *Leishmania* that were not internalized. Lymphocytes were thawed in a 37°C water bath, centrifuged at 1,200 rpm for 10 min, and adjusted to a ratio of 1 MoDC:10 lymphocytes. After this period, 3 × 10^5^ MoDCs were co-cultured with 30 × 10^5^ autologous lymphocytes for 24 h at 37°C in 5% CO_2_.

### Evaluation of the Phenotype and the Status of Activation of Dendritic Cells and Lymphocytes After Stimulation in Culture and Infection

Cultures of infected and uninfected MoDCs and lymphocytes stimulated with rSm29 (10 μg/mL) and LPS (10 ng/mL) were cultured for 24 h at 37°C, 5% CO_2_. After incubation, 20 μL/well of 10X diluted brefeldin (10 μg/mL-eBioscience) was added and a new incubation performed under the same conditions for 4 h. The MoDCs were adjusted to a count of 3 × 10^5^ and then labeled with the monoclonal antibodies CD11c-APC (clone 3.9), CD1a-FITC (clone HI149), IL-10Rα-PE (Polyclonal), CD40-PerCP-e Fluor 710 (clone 5C3), CD80-PerCP-e-Fluor 710 (clone 2D10.4), CD86-PE (clone IT2.2), CD83-PE-Cy7 (clone HB15e), and HLA-DR-PerCP-Cy5.5 (clone LN3) (all from eBioscience, California) and maintained at 4°C for 20 min. After labeling the surface molecules, the cells were centrifuged and incubated with 150 μL/well of the permeabilization buffer for 10 min at room temperature. After incubation, intracellular labeling was done using IL-12p40/70-FITC (clone 11.5), IL-10-PE (clone JES3-9D7), and TNF-PErCPCy5.5 (clone MAb11) monoclonal antibodies (all of which were from eBioscience, California) followed by incubation for 30 min at room temperature, protected from light. Then 2 washes were performed using the permeabilization buffer, followed by resuspension of the cells in 200 μL of wash solution (1x PBS + 1M Azide + BSA) and the FACS readings were performed. For the phenotypic evaluation of the lymphocytes, cell marking was performed with monoclonal antibodies against cell surface markers CD3-FITC (clone OKT3), CD4-PerCP-Cy5.5 (clone OKT-4), CD8-APC (clone OKT8), CD28-PE (clone CD28.2, ORALL), CD25-PeCy7 (clone M-A251, ORALL), and CD152 (CTLA-4)-PE (clone 14D3), all from eBioscience California, and CD40L-PeCy7 (clone 24-31; Biolegend, California). The acquisition was performed using the FACSCanto (Becton Dickinson) apparatus, for a total of 100,000 events.

Dendritic cells and lymphocytes were analyzed according to the frequency of expression of cell surface markers using the FlowJo™ program (Tree Star, USA). Cell populations were defined by non-specific fluorescence from frontal (FSC) and lateral (SSC) dispersion as cell size and granularity parameters, respectively. According to the cellular characteristics, the selection of the MoDC populations by windows in these populations was performed (Figure S3A). The frequencies of MoDC populations were made from the selection of CD11c^+^ cells (Figure S3B). T lymphocyte population analysis was performed by selecting the CD3^+^ population and the CD4^+^ and CD8^+^ subpopulations, using the Flow-Jo program (Figures S3C,D, respectively). To analyze the frequency of lymphocytes expressing IL-10, a gate was performed in a shared region of lymphocytes and the frequency of cells expressing IL-10 (Figure S3E) was evaluated.

### Determination of Cytokine Levels

Levels of the cytokines IL-10, IL-12p40, IFN-γ, and TNF were evaluated in the supernatants of MoDCs and lymphocyte co-cultures, according to the manufacturer's information (Pharmingen, San Diego, CA). The results were expressed in pg/mL.

### Microscopic Evaluation of the Infectivity of MoDCs by *Leishmania braziliensis*

To evaluate the rate of infection of MoDCs by *L. braziliensis* in the presence or absence of the rSm29 antigen, slides were prepared by cytospin (1,000 rpm, 1 min) and then the slides were stained with Panoptic stain (Interlab). The number of infected MoDCs and the number of amastigotes per 100 cells counted were evaluated. For this evaluation, 200 cells per slide were counted by two independent observers.

### Statistical Analysis

Data were analyzed using the program GraphPadPrism 5.0 (GraphPad Software, San Diego, CA, USA). Differences between MoDC frequencies and cytokine levels in patients with leishmaniasis were assessed by non-parametric ANOVA (Friedman test with Dunn's post-test). Differences between lymphocyte frequencies and infectivity among MoDCs were assessed with unpaired *T*-test. The frequencies of positive cells were expressed as medians (minimum and maximum). Cytokine concentrations were expressed as medians and standard deviations (pg/mL). Statistical significance was established at the 95% confidence interval.

## Results

### Expression of Molecules Associated With Maturation and Antigenic Presentation in Monocyte-Derived Dendritic Cells (MoDCs) Stimulated With rSm29 and Co-cultured With Autologous Lymphocytes

We evaluated the effect of the addition of the rSm29 antigen on monocyte-derived dendritic cells infected with *L. braziliensis* and co-cultured with autologous lymphocytes (MoDC-Ly) from patients with cutaneous leishmaniasis (CL).

**Figure 1:**
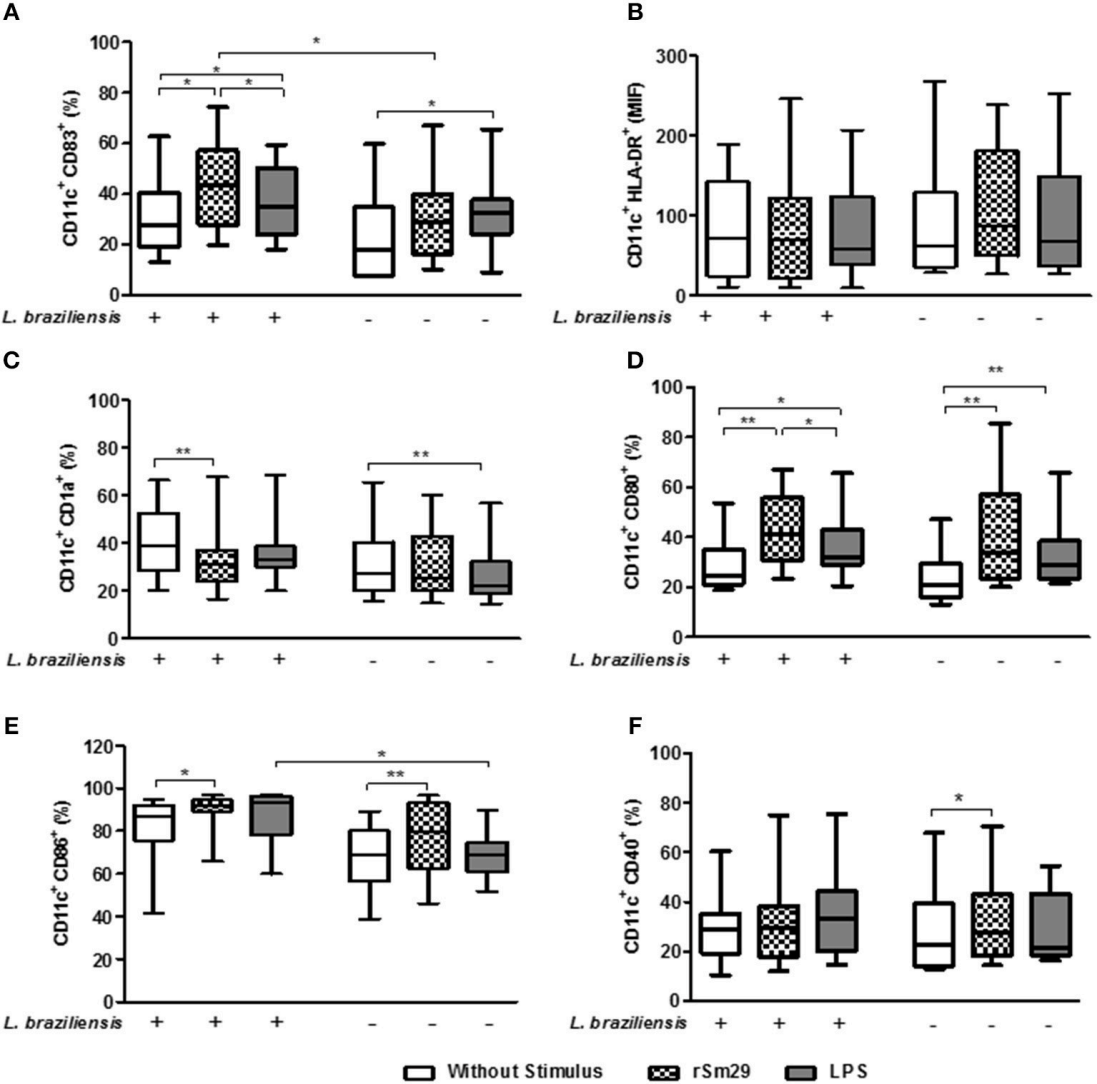
Frequency of cells expressing molecules associated with maturation [CD83 **(A)**], antigen presentation, [HLA-DR **(B)**, CD1a **(C)**], and costimulation [CD80 **(D)**, CD86 **(E)**, CD40 **(F)**] in MoDCs stimulated by rSm29 (10 μg/mL) or LPS (100 ng/mL), infected or uninfected by *L. braziliensis*, and co-cultured with autologous lymphoctyes for 24 h. The results are expressed as a median, minumum, maximum, and percentiles. *p < 0.05 and **p < 0.005. Friedman test.

In cultures infected with *L. braziliensis*, the addition of rSm29 led to an increase in the frequency of CD11c^+^CD83^+^ cells [44% (20–74)] compared to non-stimulated co-cultures [27% (13–63)] or cultures stimulated with LPS [34% (18–59), Figure 1A]. In cultures stimulated with LPS, an increase in CD11c^+^CD83^+^ cell frequency was also observed in uninfected MoDCs compared to the non-stimulated cultures from the same group (Figure 1A). We observed an increase in the frequency of CD11c^+^CD83^+^ cells in the rSm29-stimulated cultures of *L. braziliensis*-infected MoDCs [44% (20–74)] compared to uninfected cultures [29% (10–67), Figure 1A].

We also observed that the addition of the rSm29 antigen did not alter the MFI of HLA-DR in cultures of MoDCs infected with *L. braziliensis* [MFI: 87 (27–239)] or in uninfected cultures [MFI: 70 (10–246)] compared to non-stimulated cultures [infected MoDCs: 63 (28–268); uninfected MoDCs: 72 (10–189), respectively] or cultures stimulated with LPS [infected MoDCs: 68 (27–252); uninfected MoDCs: 59 (10–207), respectively, Figure 1B].

Regarding the frequency of MoDCs expressing the lipid antigen-presenting molecule CD1a, we observed a decrease in CD11c^+^CD1a^+^ frequency after stimulation with rSm29 [31% (16–68)] in the cultures infected by *L. braziliensis* compared to non-stimulated cultures [39% (20–66), Figure 1C]. In uninfected MoDC-Ly co-cultures only LPS stimulation showed a reduction in the frequency of CD11c^+^CD1a^+^ cells [22% (14–57)] when compared to non-stimulated cultures [27% (16–65), Figure 1C].

As far as the frequency of MoDCs expressing co-stimulatory molecules CD80 and CD86, it was observed that the addition of rSm29 led to an increase in the frequency of these molecules in cultures infected by *L. braziliensis* [CD80: 41% (23–67); CD86: 92% (66–97)] compared to non-stimulated cultures [CD80: 25% (19–54); CD86: 84% (41–95), Figures 1D,E, respectively]. In these cultures, stimulation of MoDCs with LPS also led to an increase in the frequency of CD80 expressing cells [32% (21–66)] compared to the non-stimulated cultures [25% (19–54), Figure 1D].

In uninfected co-cultures the addition of the rSm29 antigen also led to an increase in the frequency of CD11c^+^CD80^+^ and CD11c^+^CD86^+^ when compared to non-stimulated cultures (Figures 1D,E, respectively]. In uninfected cultures, MoDC stimulation with LPS also led to an increase in the frequency of CD80-expressing cells compared to non-stimulated cultures (Figure 1D). We also observed that in the cultures stimulated with LPS, there was an increase in CD11c^+^CD86^+^ frequency in the infected MoDCs compared to the uninfected MoDCs (Figure 1E).

Regarding, the frequency of CD11c^+^ expressing CD40, the addition of rSm29 led to an increase in the expression of this molecule only in co-cultures not infected by *L. braziliensis* compared to non-stimulated cultures (Figure 1F). Figure S4 shows the frequency of these molecules in MoDCs infected or not by *L. braziliensis* without addition of autologous lymphocytes.

Our findings demonstrate that in cultures stimulated with rSm29, infection of MoDCs with *L. braziliensis* was not able to diminish the activation profile of these cells.

### Frequency of MoDCs Expressing the Intracellular Cytokines IL-10, IL-12, and TNF When Co-cultured With Autologous Lymphocytes

**Figure 2:**
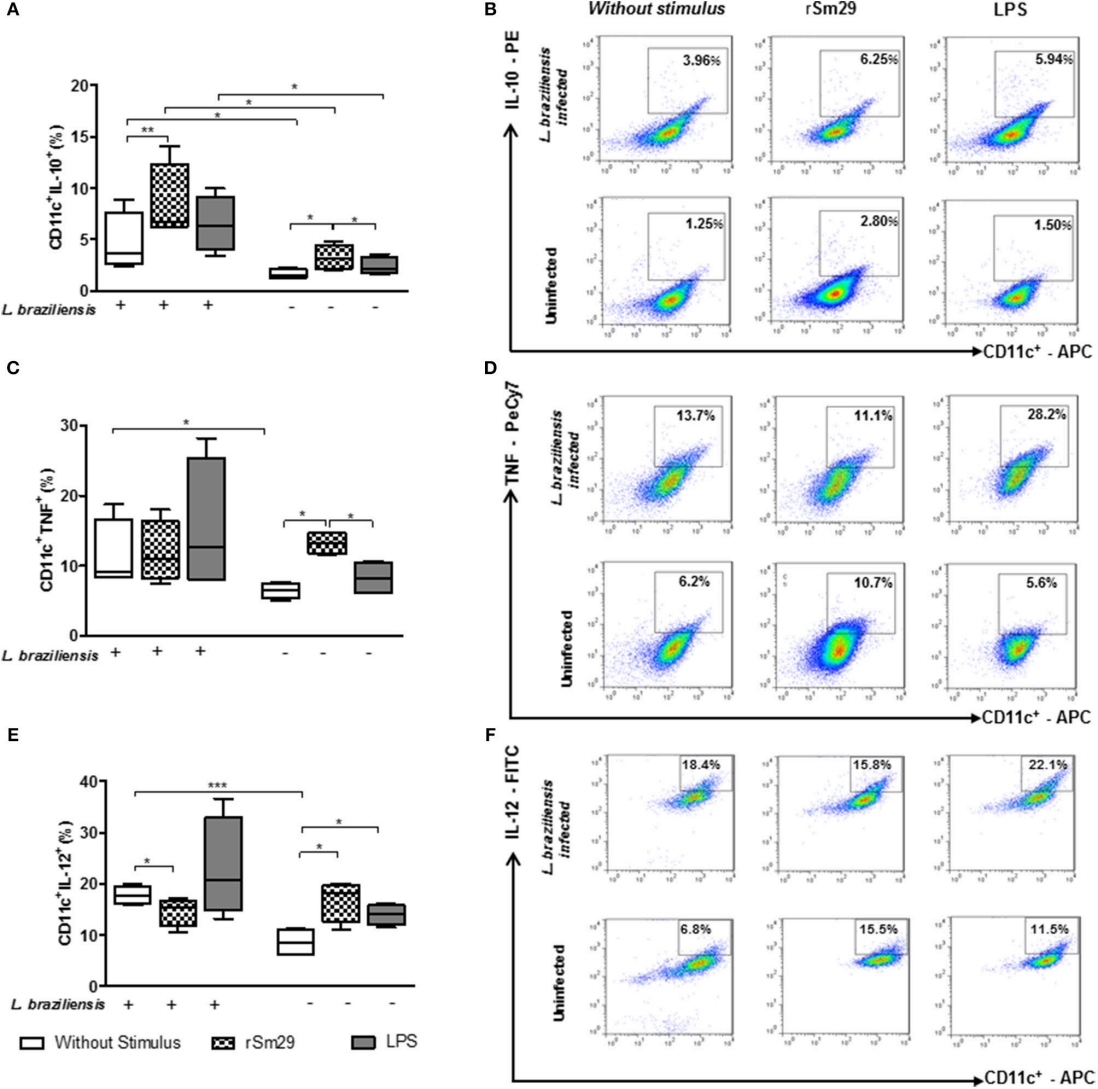
Frequency of MoDCs expressing the intracellular cytokines CD11c^+^IL-10^+^ **(A)**, CD11c^+^TNF^+^ **(B)**, and CD11c^+^IL-12^+^ **(C)** after 24 h of co-culture with autologous lymphocytes infected or uninfected by *L. braziliensis* and stimulated by rSm29 (10 μg/mL) or LPS (100 ng / ml). Representative plots of one experiment showing the frequency of CD11c^+^ cells expressing IL-10 **(D)**, TNF **(E)** and IL-12 **(F)**. Results are expressed as a median, minimum, maximum, and percentiles. *p < 0.05, **p < 0.005, and ***p < 0.001. Friedman test.

Regarding the frequency of MoDCs expressing IL-10 in *L. braziliensis*-infected co-cultures, we observed that addition of rSm29 was able to increase the frequency of CD11c^+^IL-10^+^ [6.7% (6.2–14)] compared to non-stimulated cultures [3.6% (2.4–8.9), Figures 2A,B]. When we evaluated IL-10 in uninfected MoDCs we found that the addition of rSm29 also led to an increase in the frequency of cells expressing IL-10 [3.1% (2.0–4.8)] compared to non-stimulated cultures [1.5% (1.2–2.2)] or cultures stimulated with LPS [2.2% (1.6–3.6), Figure 2A]. In infected cultures, the frequency of cells expressing IL-10 was higher either with no stimulus, stimulation with rSm29, or stimulation with LPS relative to corresponding uninfected cultures (Figures 2A,B).

Evaluating the frequency of MoDCs expressing the inflammatory cytokine TNF in *L. braziliensis*-infected cultures, we observed that the addition of rSm29 did not alter the frequency of CD11c^+^TNF^+^ compared to non-stimulated cultures or cultures stimulated with LPS (Figures 2C,D). When testing TNF in uninfected cultures we found that the addition of rSm29 was able to increase the frequency of TNF expressing cells [13% (12–15)] compared to non-stimulated cultures [7% (5–8)] or cultures stimulated with LPS [8% (6–16), Figure 2C). Non-stimulated cultures infected by *L. braziliensis* showed a higher frequency of CD11^+^TNF^+^ cells compared to uninfected cultures (Figure 2C).

Concerning the frequency of MoDCs expressing the proinflammatory cytokine IL-12, in co-cultures infected by *L. braziliensis* we observed that the addition of rSm29 decreased the frequency of CD11c^+^IL-12^+^ [15% (10–17)] compared to non-stimulated cultures [18% (16–20), Figure 2E]. On the other hand, in infected cultures we observed an increase in MoDCs expressing IL-12 when stimulated by rSm29 [18% (11–20)] or LPS [14% (11–16)] compared to non-stimulated cultures [8% (6–11), Figure 2E]. As observed with the frequency of cells expressing IL-10 or TNF, the frequency of CD11c^+^IL-12^+^ cells was greater in non-stimulated cultures infected with *L. braziliensis* compared to those cultures that were not infected (Figures 2E,F).

### Frequency of MoDCs Expressing IL-10 Receptor After Stimulation by rSm29 and Co-cultured With Autologous Lymphocytes

**Figure 3:**
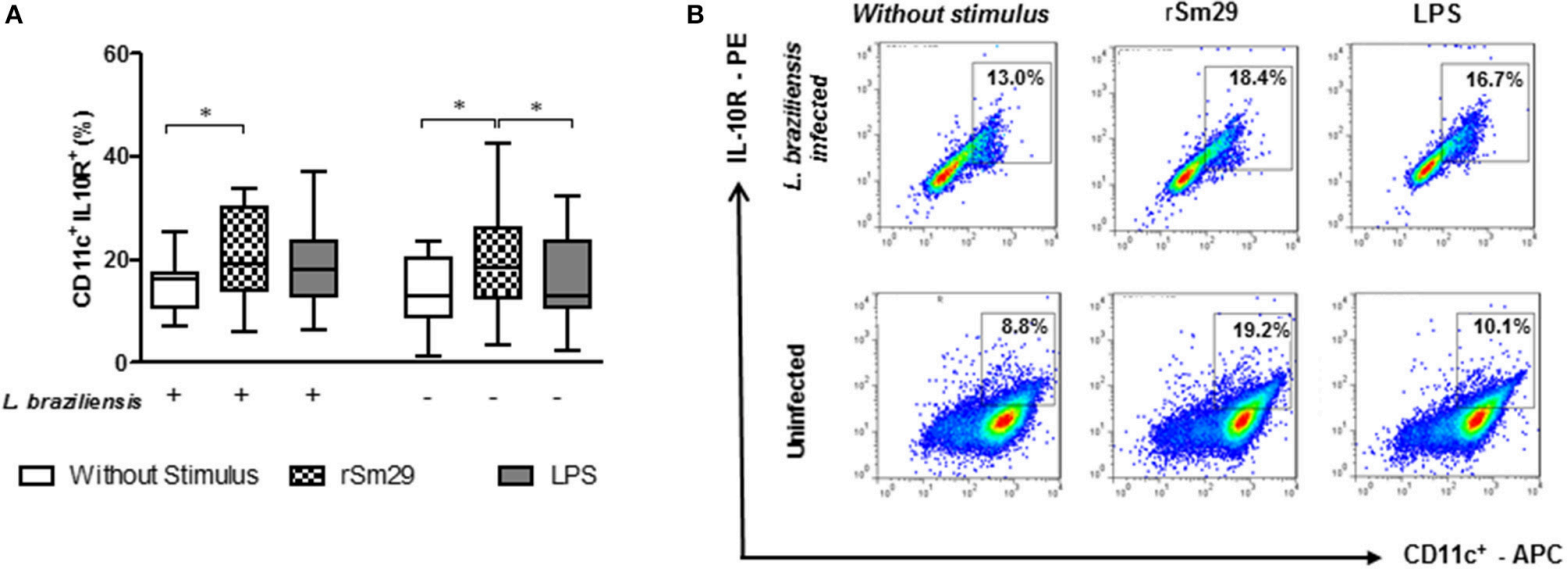
Frequency of MoDCs stimulated by rSm29 (10 μg/mL) or LPS (100 ng/mL), infected or uninfected by *L. braziliensis*, and co-cultured with autologous lymphocytes for 24 h expressing the IL-10 receptor (IL-10R). **(A)** Frequency of CD11c^+^IL-10R^+^ in MoDCs. **(B)** Representative plot of one experiment. Results are expressed as a median, minimum, maximum, and percentiles. *p < 0.05. Friedman's test.

Since the decrease in the expression frequency of the IL-10 receptor (IL-10R) *in situ* has been associated with an escalation in the inflammatory process in cutaneous lesions (10), we decided to evaluate whether rSm29 increases the frequency of CD11^+^IL-10R^+^ in co-cultures of patients with leishmaniasis infected by *L. braziliensis* (Figure 3).

We observed that the addition of rSm29 led to an increase in the frequency of cells expressing IL-10R in *L. braziliensis*-infected MoDC co-cultures from patients with CL [19% (6–34)] compared to non-stimulated cultures [16% 7–25); Figure 3A]. The same was observed in uninfected cultures, where the addition of rSm29 also increased the frequency of MoDCs expressing IL-10R [18% (3–43)] compared to non-stimulated cultures [13% (1–23)] or cultures stimulated with LPS [13% (2–23), Figure 3A]. The representative plots are shown in Figure 3B.

### Phenotypic Evaluation of CD4^+^ and CD8^+^ T Lymphocytes Co-cultured With MoDCs From Individuals With CL After Stimulation With rSm29

The phenotypic profile of the molecules associated with activation/regulation of CD4^+^ T lymphocytes in the co-cultures of infected and uninfected MoDCs and autologous lymphocytes from patients with CL was also evaluated.

**Figure 4:**
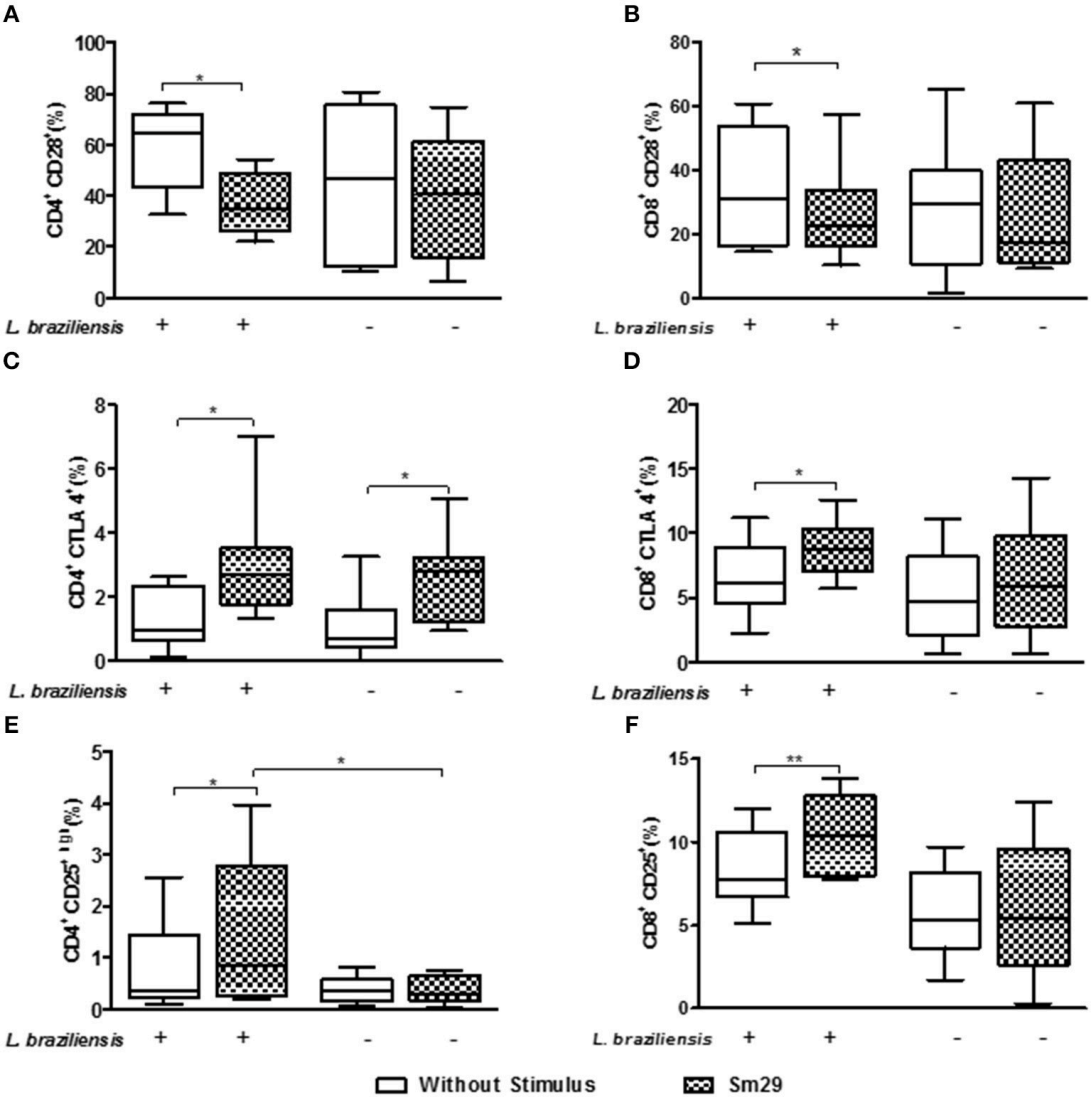
Frequency of CD4^+^CD28 **(A)**, CD8^+^CD28 **(B)**, CD4^+^CTLA-4 **(C)**, CD8^+^CTLA-4 **(D)**, CD4^+^CD25^high^ **(E)**, and CD8^+^CD25^+^ **(F)** T lymphocytes co-cultured for 24 h with MoDCs from individuals with CL after stimulation by rSm29. Results are expressed as a median, minimum, maximum, and percentiles. *p < 0.05 and **p < 0.005. Unpaired *t*-Test.

Regarding CD28, the co-stimulatory molecule associated with cell activation, the addition of rSm29 antigen led to a decrease in the frequency of CD4^+^CD28^+^ T lymphocytes [35% (22–72)] and CD8^+^CD28^+^ T lymphocytes CD28 [23% (10–58)] compared to non-stimulated cultures [CD4^+^CD28^+^:56% (17–77) and CD8^+^CD28^+^:31% (15–61), Figures 4A,B, respectively]. In uninfected cultures no statistical difference was observed with the addition of rSm29 (Figures 4A,B).

In relation to the molecules associated with regulation of the immune response we evaluated the frequency of CD4^+^ and CD8^+^ T lymphocytes expressing CTLA-4 and CD25. It was observed that the addition of rSm29 increased the frequency of CD4^+^CTLA-4^+^ [2.7% (1.3–7.0)] and CD8^+^CTLA-4^+^ T lymphocytes [9% (6–13)], compared to the non-stimulated cultures [CD4^+^CTLA-4^+^: 0.9% (0.1–2.6) and CD8^+^CTLA-4: 6% (2–11), Figures 4C,D, respectively]. The frequency of CD4^+^CTLA-4^+^ T lymphocytes was also higher in uninfected cultures [2.8% (0.9–5.1)] than non-stimulated cultures [0.7% (0.1–2.5), Figure 4C]. We also observed a higher frequency of CD4^+^CD25^hi^ [0.8% (0.2–4)] and CD8^+^CD25^+^ T lymphocytes [10% (8–14)] in co-cultures of MoDCs-Ly, relative to the non-stimulated cultures [CD4^+^CD25^hi^: 0.3% (0.1–2.6) and CD8^+^CD25^+^: 7% (5–12), Figures 4E,F, respectively].

### Levels of Cytokines in the Supernatants of MoDC-Ly Co-cultures Infected or Uninfected by *L. braziliensis*

**Figure 5:**
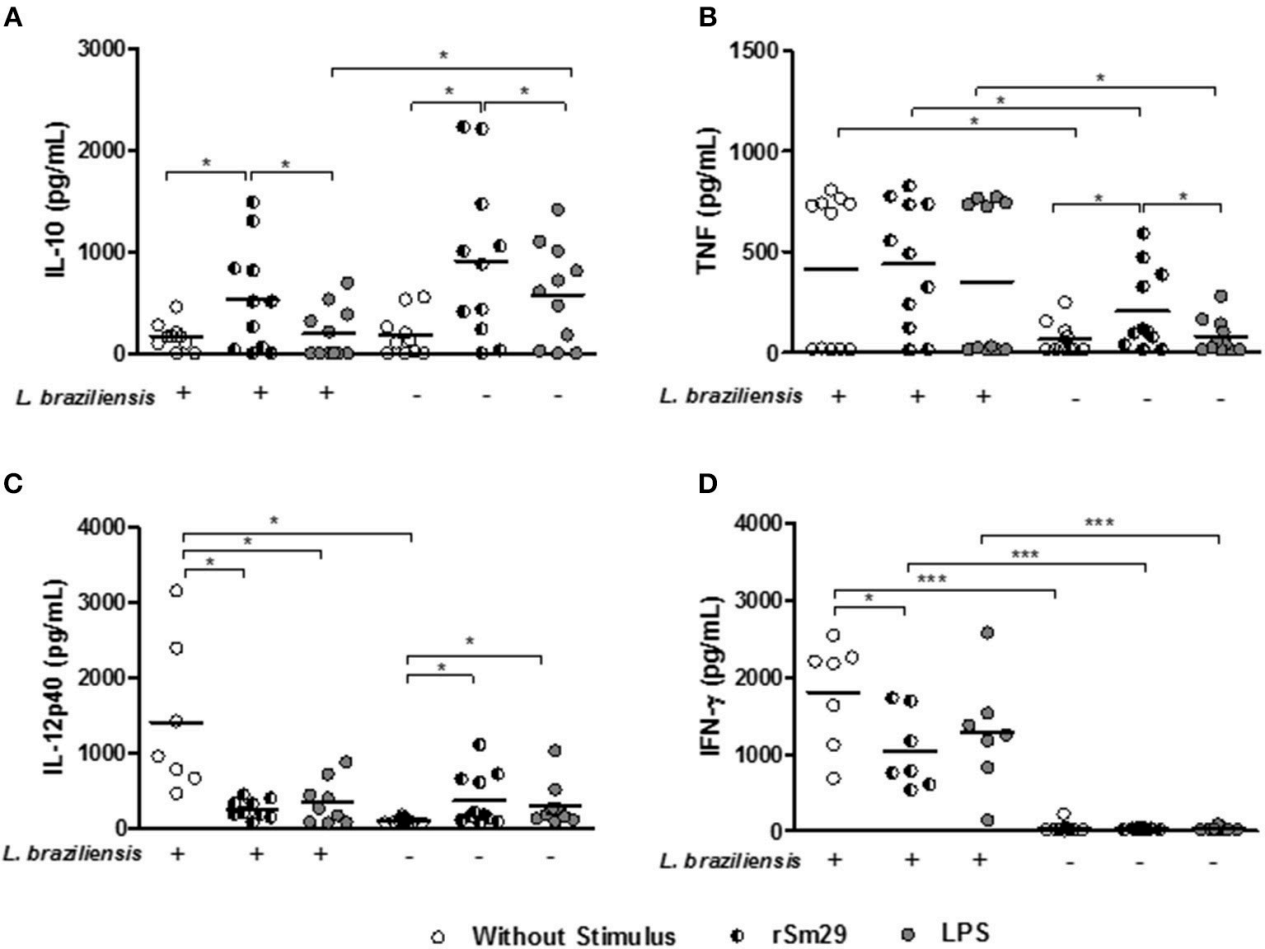
Levels of IL-10 **(A)**, TNF **(B)**, IL-12p40 **(C)**, and INF-γ **(D)** in supernatants of cultures of MoDCs stimulated or un-stimulated by rSm29, infected or uninfected by *L. braziliensis*, and co-cultured with autologous lymphocytes for 24 h. Results are expressed as a median ± standard deviation. *p < 0.05 and ***p < 0.001. Friedman test.

Herein, we also evaluated the levels of cytokines in the supernatants of MoDC-Ly co-cultures infected or uninfected with *L. braziliensis*. In supernatants of cultures stimulated with rSm29 we observed an increase in IL-10 levels (536 ± 527 pg/mL) relative to non-stimulated cultures (171 ± 134 pg/mL) or cultures stimulated by LPS [(202 ± 253 pg/mL), Figure 5A]. On the other hand, in these infected cultures, there was a decrease in IL-12p40 (257 ± 124 pg/mL) and IFN-γ (1044 ± 501 pg/mL) levels compared to non-stimulated cultures [IL-12p40: 1126 ± 1029 pg/mL; *p* < 0.05; IFN-γ: 1,810 ± 684 pg/mL). There was no change in TNF levels with the addition of rSm29 to cultures infected by *L. braziliensis* (Figure 5B). Infected cultures stimulated with LPS showed lower levels of IL-12p40 (376 ± 292 pg/mL) compared to non-stimulated cultures (IL-12p40: 1,126 ± 1,029 pg/mL, Figure 5C).

Since IL-10 is a key cytokine in the control of an exaggerated immune response, and we observed that Sm29 induces the production of this cytokine by MoDCs, we decided to investigate whether lymphocytes could contribute to the production of IL-10 seen in the culture supernatant (Figure S5). We observed a higher frequency of lymphocytes expressing IL-10 in the Sm29-stimulated cultures compared to the non-stimulated cultures. In non-infected MoDC-Ly cultures there was no difference in the expression of this cytokine by lymphocytes.

In uninfected MoDC-Ly co-cultures we observed that the addition of rSm29 was able to increase levels of IL-10 (913 ± 794 pg/mL), TNF (204 ± 203 pg/mL), and IL-12p40 (369 ± 248 pg/mL) relative to non-stimulated cultures [IL-10: 499 ± 1,054 pg/mL; TNF: 65 ± 78 pg/mL; IL-12p40: 100 ± 33 pg/mL, Figures 5A–C] or cultures stimulated with LPS [IL-10: 583 ± 487 pg/mL; TNF: 80 ± 86 pg/mL, Figures 5A,B]. Also, in these cultures LPS stimulation led to an increase in IL-12p40 levels (295 ± 270 pg/mL) compared to the non-stimulated cultures (100 ± 33 pg/mL, Figure 5C). In this group, IFN-γ levels were similar between cultures stimulated with rSm29 (37.7 ± 11.9 pg/mL), with LPS (49.8 ± 60.0 pg/mL), or not stimulated at all [(41.0 ± 22.2 pg/mL); Figure 5D].

Infection of MoDCs by *L. braziliensis* in non-stimulated cultures increased levels of TNF, IL-12p40, and IFN-γ compared to uninfected cultures (Figures 5B–D, respectively).

Regarding IL-4 levels, we observed no differences between both infected and uninfected cultures in the presence of Sm29 in relation to the non-stimulated cultures. Regarding levels of IL-17, infected cultures without stimulation, or stimulated with either Sm29 or LPS showed higher levels of this cytokine in relation to uninfected cultures. As observed with IL-4, the addition of Sm29 did not alter IL-17 levels relative to non-stimulated cultures (not shown).

### Evaluation of MoDC Susceptibility to *Leishmania* Infection After Stimulation With rSm29

**Figure 6:**
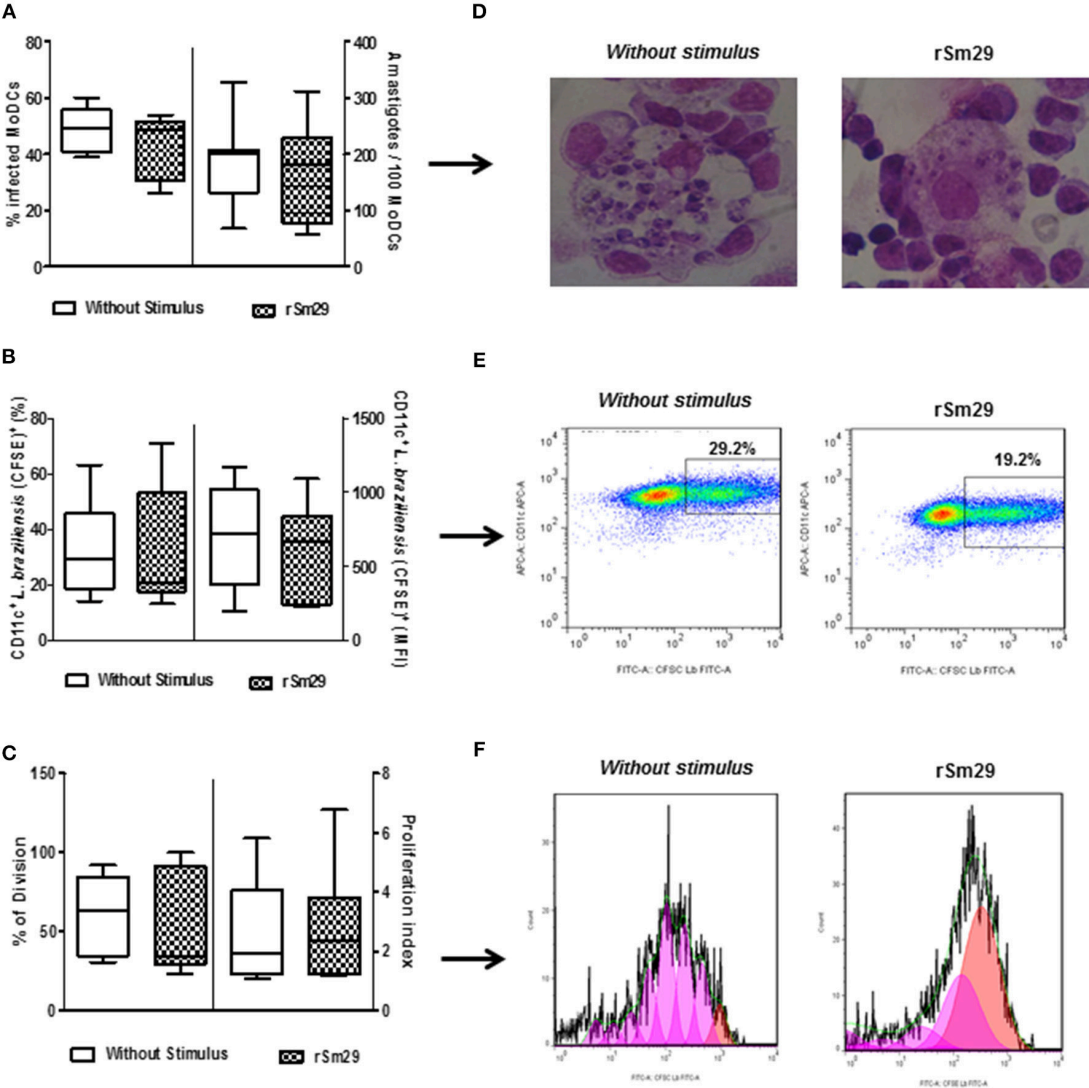
Infectivity after 2 h of *L. braziliensis* in MoDCs stimulated by rSm29 (10 μg/mL) and then co-cultured with autologous lymphocytes for 24 h. **(A)** Frequency of MoDCs infected and number of amastigotes/100 MoDCs. **(B)** Frequency of CFSE expression in *L. braziliensis* and mean fluorescence intensity of CFSE-labeled *L. braziliensis* in MoDCs (CD11c^+^). **(C)** Frequency of division of amastigotes in MoDCs and index of proliferation of amastigotes in MoDCs. **(D)** Visual representation of an experiment evaluating the infectivity of *L. braziliensis* in MoDCs. **(E)** Dot plot representing an experiment evaluating the infection rate of *L. braziliensis* (CFSE^+^) in MoDCs (CD11c^+^). **(F)** Representative histogram of an experiment evaluating proliferation of amastigotes by expression of CFSE in MoDCs (CD11c^+^). Results are expressed as a median, minimum, maximum, and percentiles.

Finally, we evaluated the effect of the addition of rSm29 antigen on the susceptibility of MoDCs to infection by *L. braziliensis*. Initially, we evaluated kinetics of infection in MoDCs by *L. braziliensis* and did not observe any difference with the addition of rSm29 compared to non-stimulated cells at 2, 24, 48, and 72 h post-infection as evaluated by optical microscopy (data not shown). In the microscopic evaluation, stimulation with rSm29 did not increase the frequency of MoDCs infected by *L. braziliensis* [39% (26–54)] or the number of amastigotes in 100 cells [220 amastigotes (57–302)] compared to non-stimulated cultures [44% (39–50); 203 amastigotes (68–328), respectively; Figures 6A,D].

The addition of rSm29 did not alter the proliferation of the *Leishmania* within the MoDCs (21%). Using MFI and CFSE as indicators of *Leishmania* proliferation in MoDCs (CD11c^+^) we observed that, just as was seen with optical microscopy, the presence of the rSm29 antigen did not alter the proliferation of the parasite within the MoDCs [21%, (13–71); MFI: 663 (222–1089)] relative to non-stimulated cultures [29% (14–63); MFI: 721 (192–1172); Figures 6B,E]. This data can be verified by Figure 6C showing the frequency of division (FD) and proliferation index (PI) of cells stimulated by rSm29 [FD: 35% (23–100); IP: 2.3 (1.2–6.8)] or not stimulated [FD: 64% (30–92); IP: 2.0 (1.1–5.8); Figures 6C,F].

## Discussion

Cutaneous leishmaniasis is due to overactivation of the predominantly Th1/inflammatory type of immune response. While the production of IFN-γ and TNF are important for elimination of *Leishmania*, it is associated with tissue injury when overactivated (4). Therefore, the cellular environment associated with proinflammatory and anti-inflammatory cytokines is important to control parasite multiplication without causing damage to the host (25, 26). In the present study we showed that *S. mansoni* rSm29 antigen induced a regulatory profile in both monocyte derived dendritic cells (MoDCs) and lymphocytes and thereby regulated the exaggerated inflammation observed in CL.

Little is known about the ability of helminth antigens to induce dendritic cell maturation. In our study we observed that rSm29 antigen induced cell maturation, as assessed by CD83 expression, in MoDCs infected with *L. braziliensis*. An *in vitro* study showed that the addition of *Taenia crassiceps* excretory/secretory antigen (TcES) to MoDC cultures from healthy individuals stimulated by LPS led to an increase in CD83 expression, but the presence of TcES alone did not alter the maturation state of these cells (27). In this study, we did not add LPS to the cultures stimulated with rSm29; however, in cultures stimulated only with LPS, we observed an increase in the maturation of these cells relative to the cultures without stimulus whether infected or not.

Regarding the expression of HLA-DR, a molecule associated with antigen presentation, we observed that stimulation by rSm29 or LPS did not change the mean fluorescence intensity of this molecule in the MoDCs infected or uninfected by *L. braziliensis* when compared to non-stimulated cultures. Previous data from our group showed that rSm29 stimulation in MoDC cultures from patients with CL yielded HLA-DR expression similar to non-stimulated cells (22). On the other hand, monocytes from CL patients stimulated with rSm29 had lower HLA-DR expression compared to non-stimulated cells (21).

Furthermore, we evaluated the influence of rSm29 antigen on the frequency of MoDCs expressing CD1a, the molecule associated with the presentation of lipid antigens. We observed that in cultures infected with *L. braziliensis* stimulation by the rSm29 antigen led to a reduction in the frequency of MoDCs expressing CD1a. It has been demonstrated that *in vitro* stimulation with helminth-derived antigens or helminth infection itself are able to decrease CD1a expression in MoDCs from both helminth-infected and healthy controls (28, 29). Therefore, the decrease in the expression of this molecule observed by stimulation with rSm29 in MoDCs infected by *L. braziliensis* could interfere with the ability to recognize lipid antigens, but it cannot be inferred if this reduction in T cell antigen presentation impairs the regulation of the immune response.

We know that antigenic co-stimulation is essential for the induction of a complete immune response. The state of activation along with the expression of costimulatory molecules of dendritic cells is important to the stimulation of T lymphocytes to assemble an effector immune response. In our study, we observed that stimulation by rSm29 led to an increase in the frequency of CD11c^+^ cells expressing both CD80 and CD86 compared to non-stimulated cultures, infected or not with *L. braziliensis*.

Different pathogens may influence the maturation and activation of dendritic cells differently and thus affect the outcome of the infection. Mice co-infected with *Schistosoma japonicum* and *Plasmodium berghei* had lower frequency of BMDCs expressing CD86 than BMDCs from *P. berghei*-infected mice (30). On the other hand, in MoDCs from healthy individuals, it was demonstrated that neither the presence of *L. amazonensis* or SLA stimulation altered the expression of CD80 and CD86 molecules when compared to DCs not cultured with the parasite or stimulated with SLA (31). The conflicting data observed in the literature could be associated with the study models, the *Leishmania* and helminth species used, or the type of dendritic cell studied.

In our study the antigen did not increase the frequency of MoDCs infected with *L. braziliensis* expressing CD40. This fact may be responsible for the lack of induction of the proinflammatory cytokines, IL-12, and TNF, observed in the study. On the other hand, in the uninfected cultures, where an increase in the frequency of CD11c^+^CD40^+^ cells after stimulation by rSm29 was observed, an increase in the frequency of the cells expressing IL-12p40 and TNF was also observed. The interaction of CD40 on human dendritic cells infected with *L. major* with CD40L on lymphocytes provides a signal for cellular activation and production of proinflammatory cytokines, such as IL-12, which induces a Th1 profile (32).

The pattern of cytokines produced in the course of leishmaniasis is central to the clinical outcome of the disease. In assessing the intracellular expression of cytokines in MoDCs, we observed in infected cultures that the addition of rSm29 led to a decrease in the frequency of cells expressing IL-12 and an increase in the frequency of these cells expressing IL-10. In these cultures there was no change in the expression of TNF. Similar to what was observed with the intracellular expression of cytokines in the MoDCs, in the supernatant of MoDCs infected with *L. braziliensis* and co-cultured with lymphocytes, stimulation by rSm29 led to an increase in IL-10 regulatory cytokine production and a decrease in the production IL-12p40 and INF-γ, without altering TNF levels. Although TNF and INF-γ are important for activation of infected macrophages and parasitic intracellular elimination, when over-expressed they are associated with increased inflammation and generation of tissue damage in CL (3, 12). Thus, maintenance of appropriate TNF levels is likely required for parasitic control.

In uninfected cultures, there was an increase in the frequency of MoDCs expressing IL-12p40, TNF, and IL-10 following stimulation by rSm29. A likely explanation for this result is the significantly higher induction of IL-10 in infected cultures compared to uninfected cultures that was potentially able to modulate IL-12p40 production only in the infected cultures.

It has been demonstrated, not only in leishmaniasis but also in other disease models that stimulation *in vitro* with rSm29 results in enhanced production of IL-10 (33–35). This was the rationale for choosing this antigen. IL-10 is an important cytokine with anti-inflammatory and immunosuppressive activities.

Unlike *S. mansoni* egg antigens that are important inducers of a Th2 response profile with enhanced production of IL-4 (36–38), antigens located in the tegument, likely because they are in constant exposure to the cells of the immune system, are for the most part inducers of a potent regulatory response mainly characterized by IL-10 production. This appears to be a mechanism of parasite survival in host tissues. The native protein Sm29 is present in the tegument of the adult worm and our group's works have demonstrated its ability to induce a regulatory immune response mainly with the production of IL-10. In PBMCs from individuals with CL stimulated with SLA, the Sm29 antigen induces a Treg-like response with IL-10 production but without increasing the Th2-type response (20). Similar findings were observed in PBMCs from patients with asthma, either infected or uninfected by *S. mansoni*, where the addition of Sm29 antigen led to an increase in the Treg response with IL-10 production and no increase in the Th2 response (39).

The cytokine IL-10 has been also associated with regulation of expression of HLA-DR and costimulatory molecules (40). Activation of DCs isolated from the spleen of IL-10-deficient mice was compared to the same cells from animals infected with *S. mansoni* and found that in these deficient animals there was an increase in HLA-DR, CD80, CD86, and CD40 expression, suggesting that maturation and activation of DCs was regulated by the production of IL-10 (33). In this study the IL-10 induced by rSm29 in the MoDCs and lymphocytes may be limiting the expression of some activation molecules and costimulatory molecules in MoDCs. This unconventional activation of MoDCs may favor a balance between an effector response against *Leishmania* and a protective regulatory response against exaggerated imflammation.

Our study also showed that the frequency of cells expressing the IL-10 receptor (IL-10R) is increased in the rSm29-stimulated co-cultures, both in the MoDCs infected and not infected by *L. braziliensis*. Previously, we had demonstrated that MoDCs of individuals with CL stimulated with rSm29 and SLA had increased IL-10R expression compared to non-stimulated cells, cells stimulated only with SLA, or cells stimulated with rSm29 alone (22). A study published by Faria et al. showed that IL-10R expression deficiency *in situ* in the lesion would be associated with the exaggerated response observed in mucosal leishmaniasis when compared to CL (41). The increase of IL-10R in MoDCs may contribute to a regulation of these cells and consequently less activation of the inflammatory response responsible for cutaneous lesions in leishmaniasis.

In this study, we further evaluated whether stimulation by rSm29 changes the phenotypic profile of CD4^+^ and CD8^+^ T lymphocytes in the MoDC co-cultures of patients with CL infected or not infected by *L. braziliensis*. We observed a decrease in CD4^+^ and CD8^+^ T lymphocytes expressing CD28 in the cultures stimulated with rSm29, as well as an increase in the frequency of these lymphocytes expressing the cell-associated regulatory molecules, CTLA-4 and CD25. In *Schistosoma* infection, natural Treg cells (CD4^+^CD25^+^) and, to a lesser extent, Th2 cells play roles in the suppression of Th1 responses during schistosomiasis (42). Mice coinfected with *S. japonicum* and *P. berghei* showed a higher frequency of Treg lymphocytes compared to mice infected with *Plasmodium* alone (30).

Studies from our group evaluating *S. mansoni* antigens have shown the ability of these antigens, among them rSm29, to induce the expression of regulatory molecules in T lymphocytes (21, 33–35). This could be one of the mechanisms by which we observed a decrease in the production of IFN-γ and an increase in the production of IL-10 in the supernatants of cultures stimulated with rSm29 in the present study.

Furthermore, our study demonstrated that the rSm29 antigen did not increase the infectivity of *L. braziliensis* in MoDCs as measured by both the frequency of infected cells and the number of internalized amastigotes. Some studies indicate that *S. hematobium* coinfection promotes some protection against *Plasmodium falciparum* infection, due to low parasitemia seen in evaluated children (43).

In the evaluation of CFSE-labeled *L. braziliensis* by flow cytometry there was no difference in frequency and MFI of CFSE in MoDCs (CD11c^+^) upon addition of rSm29. These results suggest that the proliferation of amastigotes within the MoDCs was not influenced by this antigen. Therefore, the non-proliferation of this parasite in MoDCs stimulated by rSm29 was an important finding, suggesting an alteration in the immune response controlled mainly by the induction of IL-10 and reduction of IL-12, without a deactivation of the effector response. In addition, macrophages are the effector cells responsible for intracellular destruction of the parasite and, in this context, TNF plays a key role in the control of disease. The presence of this inflammatory cytokine in the cultures stimulated by rSm29 may aid in the maintenance of this macrophage activity without, however, favoring the over activation of the inflammatory response.

There are no data in the literature on the induction of leishmanicidal effector mechanisms by dendritic cells. Dendritic cells (DCs) are recognized for their ability to sensitize naïve T lymphocytes and for contributing to the functional differentiation of regulatory T cells (7), as well as being important sources in the production of cytokines and the presentation of parasite antigens to T cells. However, observation that Sm29 did not increase the parasite burden is very positive suggesting that it despite reduced the immune response is it not associated with parasite multiplication.

Considering that there are few therapeutic options for CL and that lately the incidence of cases refractory to conventional treatment has increased (44, 45), the use of rSm29 may be an alternative to current treatment and may be an important strategy to reduce the healing time of lesions in patients with CL caused by *L. braziliensis*.

In conclusion the rSm29 antigen was able to increase the *in vitro* expression of activation markers in MoDCs, the expression of regulatory molecules associated with CD4^+^ and CD8^+^ T lymphocytes, and IL-10 levels in the supernatants of MoDCs co-cultured with lymphocytes, without increasing the susceptibility to infection of MoDCs by *L. braziliensis*. Regulation of the *in vitro* immune response by this unconventional activation of MoDCs stimulated by rSm29 suggests that this antigen has the potential to induce a desirable, balanced immune response in the control of CL.

## Author Contributions

LSC, EC, and SO designed the project and experiments. DL, LPC, and BP carried out most of the experiments. DL, BP, and LSC wrote the manuscript and carried out statistical analysis and prepared figures. LSC and DL submitted this paper. All authors reviewed the manuscript.

### Conflict of Interest Statement

The authors declare that the research was conducted in the absence of any commercial or financial relationships that could be construed as a potential conflict of interest.
