# Supplementary material for: Schistosoma mansoni rSm29 Antigen Induces a Regulatory Phenotype on Dendritic Cells and Lymphocytes From Patients With Cutaneous Leishmaniasis
Source: Front Immunol. 2019 Jan 9;9:3122. doi: 10.3389/fimmu.2018.03122 (PMC6333737; doi:10.3389/fimmu.2018.03122)
Supplement: Supplementary file 1 [file Data_Sheet_1.PDF]

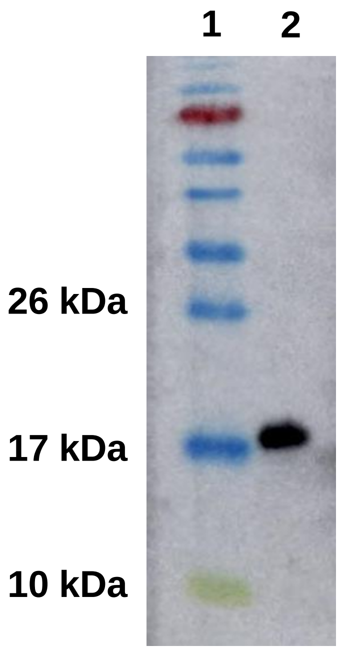


**Figure S1. Western blot analysis of purified recombinant Sm29.** The recombinant protein Sm29 was expressed in BL21 DE3 *E. coli* strain and purified by affinity chromatography. The purified content was then submitted to SDS-PAGE followed by westernblot analysis. 1. Molecular weight (PageRuller Prestained Protein Ladder; Thermo Scientific); Lane 2, rSm29.
