# Supplementary material for: Schistosoma mansoni rSm29 Antigen Induces a Regulatory Phenotype on Dendritic Cells and Lymphocytes From Patients With Cutaneous Leishmaniasis
Source: Front Immunol. 2019 Jan 9;9:3122. doi: 10.3389/fimmu.2018.03122 (PMC6333737; doi:10.3389/fimmu.2018.03122)
Supplement: Supplementary file 2 [file Data_Sheet_2.PDF]

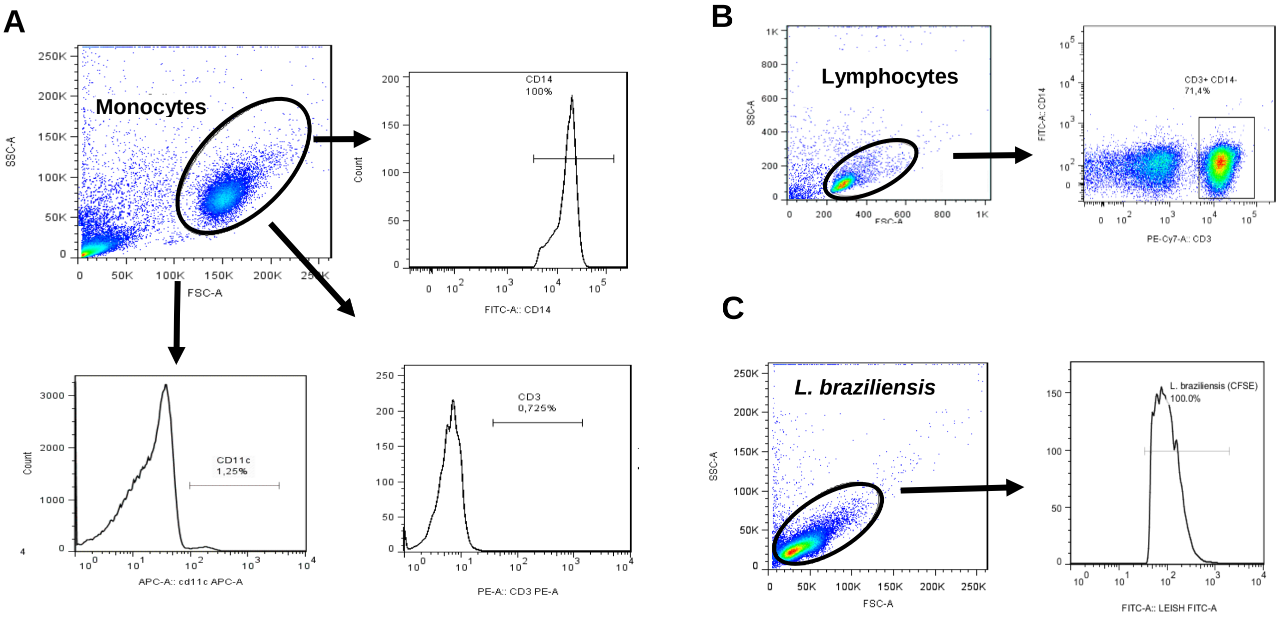

FIGURE S2. Isolation of monocytes (A) and lymphocytes (C) by magnetic beads and identification of CFSE-labeled *Leishmania braziliensis* (B).
