# Supplementary material for: Schistosoma mansoni rSm29 Antigen Induces a Regulatory Phenotype on Dendritic Cells and Lymphocytes From Patients With Cutaneous Leishmaniasis
Source: Front Immunol. 2019 Jan 9;9:3122. doi: 10.3389/fimmu.2018.03122 (PMC6333737; doi:10.3389/fimmu.2018.03122)
Supplement: Supplementary file 3 [file Data_Sheet_3.PDF]

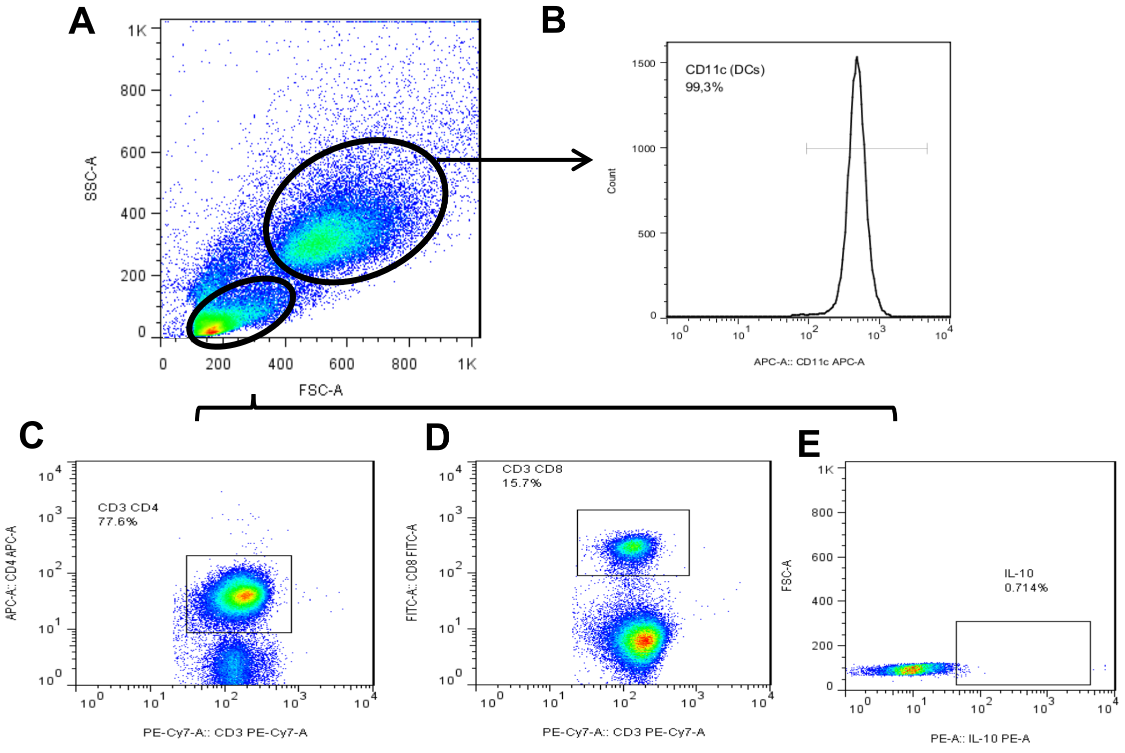

FIGURE S3. Gate strategy for the identification of monocytes-derived dendritic cells and lymphocytes. Figure A represents the selection strategy via a non-specific fluorescence densitometry plot for size (FSC) and cell granularity (SSC), identifying populations of dendritic cells (upper) and lymphocytes (lower) that were co-cultured for 24h. Figure B represents the marker of MoDCs (CD11c$^+$) evaluated within the MoDC population while Figures C and D show CD3$^+$CD4$^+$ or CD3$^+$CD8$^+$ T lymphocyte subpopulations evaluated within the lymphocyte population, respectively. Figure E represents the expression of IL-10 in non-specific lymphocytes region (FSC).
