# Supplementary material for: Schistosoma mansoni rSm29 Antigen Induces a Regulatory Phenotype on Dendritic Cells and Lymphocytes From Patients With Cutaneous Leishmaniasis
Source: Front Immunol. 2019 Jan 9;9:3122. doi: 10.3389/fimmu.2018.03122 (PMC6333737; doi:10.3389/fimmu.2018.03122)
Supplement: Supplementary file 4 [file Data_Sheet_4.pdf]

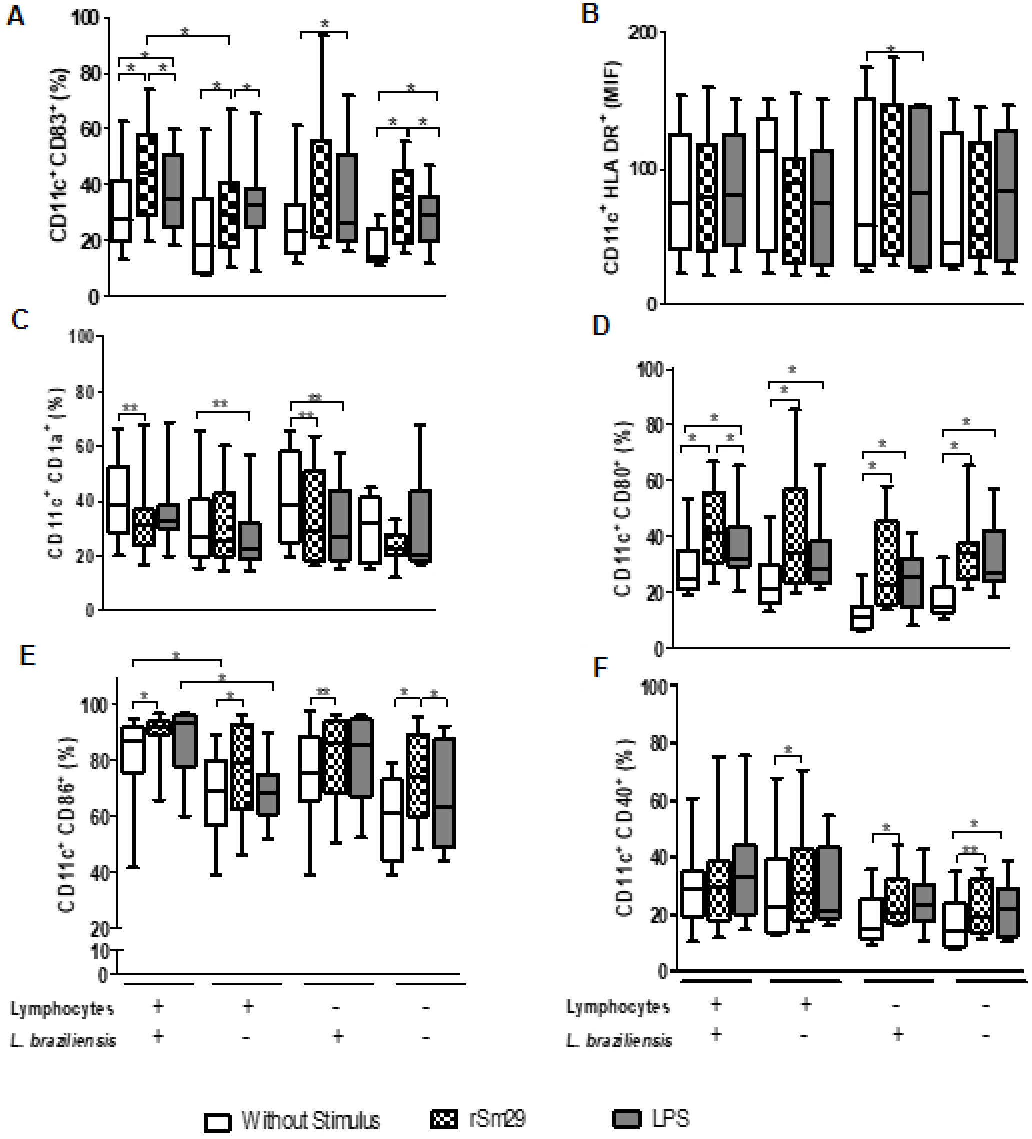

FIGURE S4. Frequency of cells expressing molecules associated with maturation [CD83 (A)], antigen presentation, [HLA-DR (B), CD1a (C)], and costimulation [CD80 (D), CD86 (E), CD40 (F)] in MoDCs stimulated by rSm29 (10µg/mL) or LPS (100ng/mL), infected or uninfected by *L. braziliensis*, and co-cultured with/without autologous lymphoctyes for 24h. The results are expressed as a median, minumum, maximum, and percentiles. *p<0.05 and **p<0.005. Friedman test.
