# Supplementary material for: Schistosoma mansoni rSm29 Antigen Induces a Regulatory Phenotype on Dendritic Cells and Lymphocytes From Patients With Cutaneous Leishmaniasis
Source: Front Immunol. 2019 Jan 9;9:3122. doi: 10.3389/fimmu.2018.03122 (PMC6333737; doi:10.3389/fimmu.2018.03122)
Supplement: Supplementary file 5 [file Data_Sheet_5.pdf]

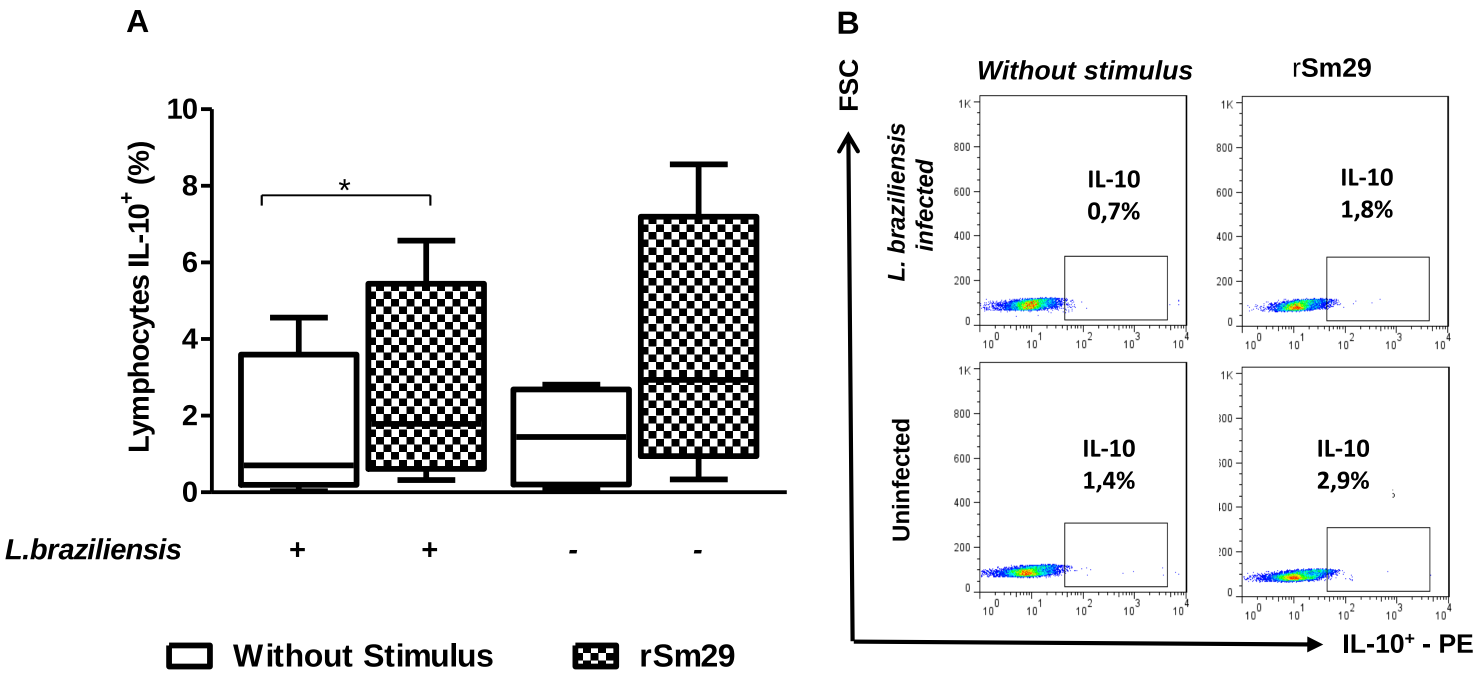

FIGURE S5. Frequency of lymphocytes expressing the intracellular IL-10 after 24h of co-culture with MoDCs infected or uninfected by *L. braziliensis* and stimulated by rSm29 (10µg/mL). Representative plots of one experiment showing the frequency of lymphocytes expressing IL-10 (B). Results are expressed as a median, minimum, maximum, and percentiles. *p<0.05, Unpaired t-Test.
